# A replication-deficient H9N2 influenza virus carrying H5 hemagglutinin conferred protection against H9N2 and H5N1 influenza viruses in mice

**DOI:** 10.3389/fmicb.2022.1042916

**Published:** 2022-11-15

**Authors:** Weigang Ren, Shuli Pei, Wenming Jiang, Meixia Zhao, Le Jiang, Honggang Liu, Yongxiang Yi, Mizhou Hui, Junwei Li

**Affiliations:** ^1^School of Life Science, Northeast Agricultural University, Harbin, China; ^2^Henan Vocational College of Agriculture, Zhongmu, China; ^3^Laboratory of Surveillance for Avian Diseases, China Animal Health and Epidemiology Center, Qingdao, China; ^4^College of Veterinary Medicine, Qingdao Agricultural University, Qingdao, China; ^5^Department of Infectious Diseases, The Second Hospital of Nanjing, Nanjing University of Chinese Medicine, Nanjing, China; ^6^The Clinical Infectious Disease Center of Nanjing, Nanjing, China

**Keywords:** replication-deficient virus vaccine, recombination, influenza A virus, H9N2, H5N1

## Abstract

H5N1 and H9N2 influenza viruses have been reported to cause human infections and are believed to have pandemic potential. The vaccine is an effective tool to prevent influenza virus infection. However, inactivated influenza vaccines sometimes result in low antigenicity as result leads to generating of incomplete immune protection in the form of low cellular and humoral immunity. While the low temperature adapted, traditional live attenuated influenza vaccine (LAIV) is associated with the potential risk to revert to a virulent phenotype, there appears an essential need for an alternative potent methodology to design and develop influenza vaccines with substantial safety and efficacy which may confer solid protection against H9N2 or H5N1 influenza virus infections. In the present study, a replication-deficient recombinant influenza virus, WM01ma-HA(H5), expressing hemagglutinin (HA) of both H9N2 and H5N1 subtypes was developed. The chimeric gene segment expressing HA(H5), was designed using the sequence of an open reading frame (ORF) of HA adopted from A/wild duck/Hunan/021/2005(H5N1)(HN021ma) which was flanked by the NA packaging signals of mouse-adapted strain A/Mink/Shandong/WM01/2014(H9N2)(WM01ma). Due to the absence of ORF of structural protein NA, the replication of this engineered H9N2 influenza viruses WM01ma-HA(H5) was hampered *in vitro* and *in vivo* but was well competent in MDCK cells stably expressing the NA protein of WM01ma. Intranasal vaccination of mice with WM01ma-HA(H5) stimulated robust immune response without any clinical signs and conferred complete protection from infection by H5N1 or H9N2 subtype influenza viruses.

## Introduction

Avian influenza viruses (AIV) target a wide variety of hosts including birds, swine, humans, and several other mammals (Proenca-Modena et al., 2007; Yoon and Webby, 2014). Few years back, H9N2 and H5N1 influenza viruses were the most prevalent subtypes in several provinces of China (Stallknecht and Shane, 1988; Olsen et al., 2006; Tong et al., 2013). Currently, different epidemiological studies indicate that H9N2 AIV(s) is present in various regions of the globe as this strain is being isolated from several animal species including duck, swine, quail, pigeon, and pheasant (Sun and Liu, 2015; Xu et al., 2015). The H5N1 subtype is a highly pathogenic avian influenza (HPAI) virus which is often considered to have pandemic potential owing to its high transmissibility and immune escape (Skeik and Jabr, 2008; Young et al., 2016). Presently, the H5N1 HPAI virus is reported to have been detected in more than 60 countries and its existence blows to the economy and industry linked with poultry (Eagles et al., 2009; Swayne, 2012; Saito et al., 2015). Some research studies suggest that H9N2 AIV(s) may have contributed to the genetic and geographic diversity of H5N1 virus(s) (Monne et al., 2013; Munir et al., 2013). In addition, it is well-studied that the H9N2 virus is competent enough to contribute internal genes to emerging influenza viruses, such as H7N9 and H10N3. This property of sharing the genetic potential facilitates the development of influenza virus vaccine candidates to prevent the possible emerging influenza virus through the reassortment of gene segment exchange (Liu et al., 2013; Wang et al., 2021).

In the current scenario, H9N2 and H5N1 influenza viruses pose a significant threat not only to the poultry industry but also to general public health, owing to the spillover from avian to mammalian species (Peiris et al., 1999; Butt et al., 2005; Ali et al., 2019). Plethora of cases have been reported that people were infected with H9N2 or H5N1 viruses due to direct contact with infected animals (Guan et al., 1999, 2000; Lin et al., 2000; Chen et al., 2009; Guan and Smith, 2013). A comprehensive surveillance study results showed H9N2 seropositivity in a huge number of poultry farm workers because of occupational exposure in China (Li et al., 2007). It is, therefore, obvious that H9 and H5 AIV subtypes pose a potential threat to human public health and place a dire need for designing a methodology to block their spread (Li et al., 2004; Butt et al., 2005; Zhao et al., 2013).

In the initial step of replication of the influenza virus, HA takes lead in performing functions, such as receptor-binding and membrane fusion. Hemagglutinin (HA) is also the predominant inducer of neutralizing antibody production to block virus infection. Apart from the recent technical and technological advancement, it appears that vaccinating poultry birds with inactivated influenza vaccine is still the key strategy to control AIV(s) infections; however, there are certain limitations lying with this strategy as the partial humoral and cellular immune response indicates that inactivated influenza vaccines fail to trigger a vigorous immune response, hence, creating a room for advanced research into a more potent vaccine development is necessary. Live attenuated influenza vaccines (LAIVs) are reported to provide strong long-lasting cell-mediated and potent humoral immunity, nevertheless, the biological safety of LAIV is a major concern for researchers and poultry farmers. The fear of virulence reversal and viral shedding by the LAIV is a key hurdle for the farmers and poultry business community to take risks for a better understanding of vaccine functions (Guo et al., 2014; Li et al., 2014; Zhou et al., 2016).

Research studies with LAIV showed that it conferred heterosubtypic immunity in animal models. Unfortunately, clinical trials have provided evidence that a licensed temperature-sensitive influenza vaccine, FluMist, shed virus postvaccination in children and adults (Straight et al., 2006; Kreijtz et al., 2007; Block et al., 2008; Hammitt et al., 2009). Keeping in view all the above-mentioned facts that there appears a dire need to design and develop novel approaches, are in order to invent safe and potent vaccines that can or may assure and elicit effective, cross-reactive, and confer long-lasting immunity.

The influenza virus genome includes eight segmented RNAs, which facilitate the reassortment and exchange of RNA segments. For the occurrence of an efficient replication, all the eight viral RNA (vRNA) segments must get incorporated into progeny virions. Segment-specific RNA packaging sequences which play a crucial role in successful packaging have been identified on each segment of influenza virus (Fujii et al., 2003; Hutchinson et al., 2010). The 3' and 5' ends of noncoding regions (NCRs) and the 183- and 157-nt of the coding region at the 3' and 5' terminals of the NA vRNA are essential to efficiently encapsulate vRNAs into progeny virions (Fujii et al., 2003). These packaging signals have been used to generate bivalent vaccines, genome-modified LAIVs, and replication-deficient recombinant influenza viruses (Gao et al., 2010; Masic et al., 2013).

In this study, a replication-deficient recombinant H9N2 influenza virus bearing H5 HA coding sequence was generated. Due to the lack of structural protein NA, the recombinant influenza virus is non-replicative both *in vitro* and *in vivo*; however, replicates well on an MDCK cell line stably expressing NA protein. Vaccination with this replication-deficient recombinant H9N2 influenza virus elicited a robust immune response which provided the host with standard protection against both H9N2 and H5N1 subtype viruses, highlighting its potential to serve as a safe bivalent vaccine candidate.

## Results

### Generation of MDCK stably expressing the NA protein of WM01ma

**Figure 1:**
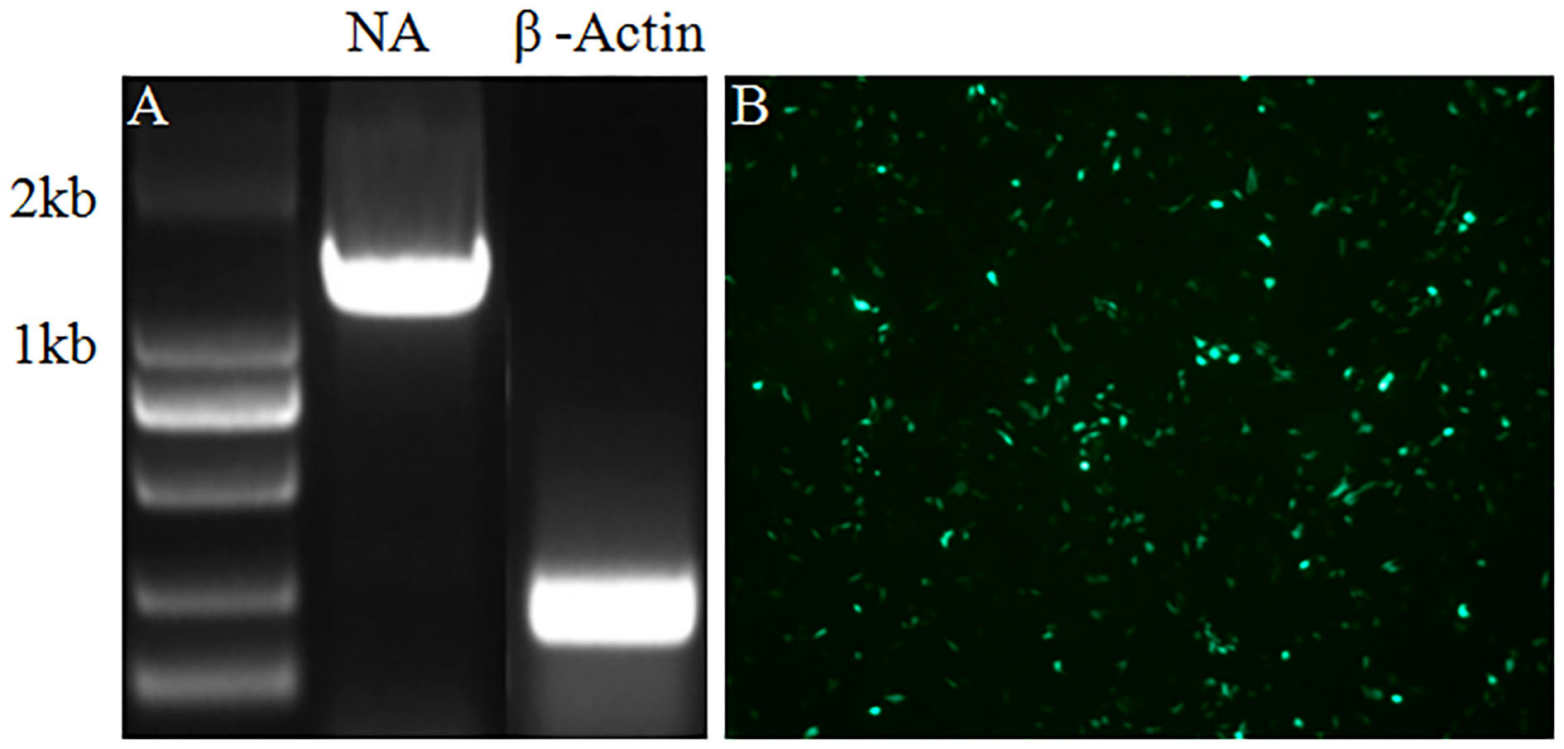
RT-PCR and IFA were performed to verify the integration and expression of the NA in MDCK cells stably expressing NA protein. **(A)** The NA gene was amplified by RT-PCR with mRNA of β-Actin as internal control. **(B)** Expression of the NA protein was tested by IFA.

In the current study, a helper cell line was established to facilitate the package of chimeric recombinant influenza virus virions. Following the standard procedure, the ORF of the NA gene was inserted into a G418 selection plasmid, and MDCK cells were transfected with a plasmid carrying the neomycin-resistance gene. Then, the transfected MDCK cells were selected in a medium with 600 μg/ml G418 by the virtue of serial passages. To verify the successful integration of the NA gene into the genome of MDCK cells and its expression, RNA of the MDCK cells showing stable expression of the NA protein was extracted and the NA ORF gene segment was amplified by reverse transcription-polymerase chain reaction (RT-PCR). As indicated in Figure 1A, the NA gene segment was amplified by RT-PCR. The result in Figure 1B indicated that the NA protein was expressed in MDCK cells stably expressing the NA protein tested by immunofluorescence assay (IFA). All-inclusive results showed that MDCK cells stably expressing the NA protein were generated by transfection and serial selection with G418.

### Generation of recombinant H9N2 influenza viruses carrying H5 subtype HA

**Figure 2:**
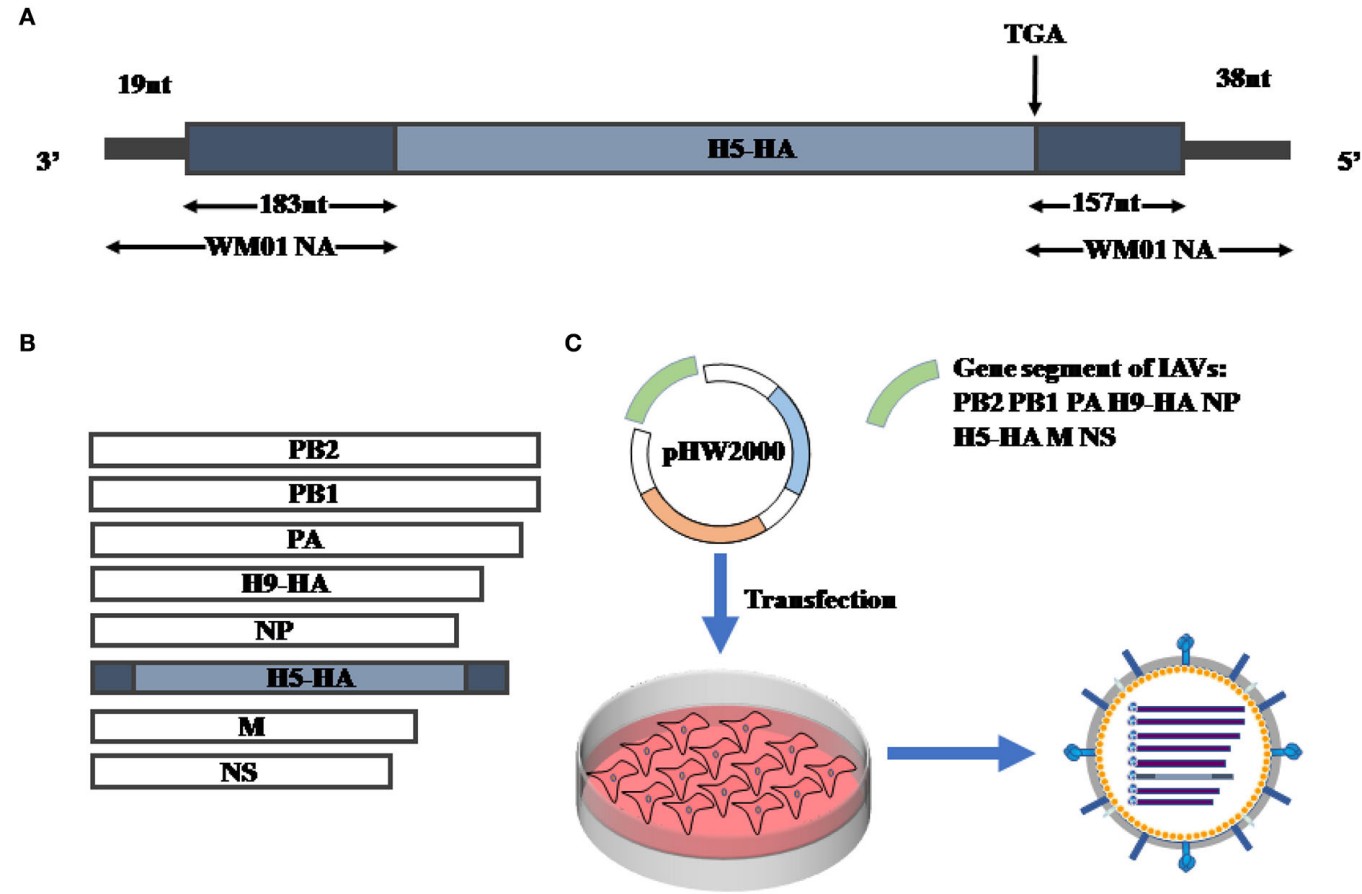
Generation of replication-deficient recombinant WM01ma-HA (H5). **(A)** Schematic representation of chimeric HA gene segment. The open reading frame (ORF) of HA (HN01ma, H5N1) is flanked by the NA segment-specific packaging sequences derived from WM01ma (H9N2). **(B)** The eight segments for WM01ma-HA (H5) recombinant viruses. **(C)** Rescue of recombinant WM01ma-HA (H5) influenza virus by the reverse genetic system.

Previous studies indicated a live-attenuated swine influenza vaccine (LASIV) candidate harboring two different HAs of H1 and H3 subtypes was generated by utilizing modified packaging signals of the NA gene segment (Masic et al., 2013; Landreth et al., 2021). Referring to the standard method, a replication-deficient recombinant influenza virus was rescued by using the genetic information of A/wild duck/HN/021/2005(H5N1)(HN021ma) (Li et al., 2009) and A/Mink/SD/WM01/2014(H9N2) (WM01ma) (Ren et al., 2020). Initially, a chimeric influenza HA(H5) gene segment was designed and synthesized flanked with the NA(H9) packaging signals at the 3' and 5' ends (202 nucleotides and 195 nucleotides, respectively) (Figure 2A). In the next step, the chimeric DNA sequence was cloned into pHW2000 (Hoffmann et al., 2000). The translation initial codon and stop codon in the packaging signals of the NA(H9) gene segment were mutated to facilitate the HA(H5) to get translated adequately. Subsequently, the designated plasmid pHW-NAps-HA-NAps along with other seven plasmids (pHW-PB2, pHW-PB1, pHW-PA, pHW-HA, pHW-NP, pHW-M, and pHW-NS) coding gene segments of WM01ma (H9N2) influenza virus were co-transfected into 293T cells and MDCK cells which were stably expressing the H9N2 NA protein. In the final step, the recombinant swap virus was successfully rescued and named WM01ma-HA(H5) virus (Figures 2B,C).

### Characterization of the H9N2 influenza virus carrying H5 subtype HA

**Figure 3:**
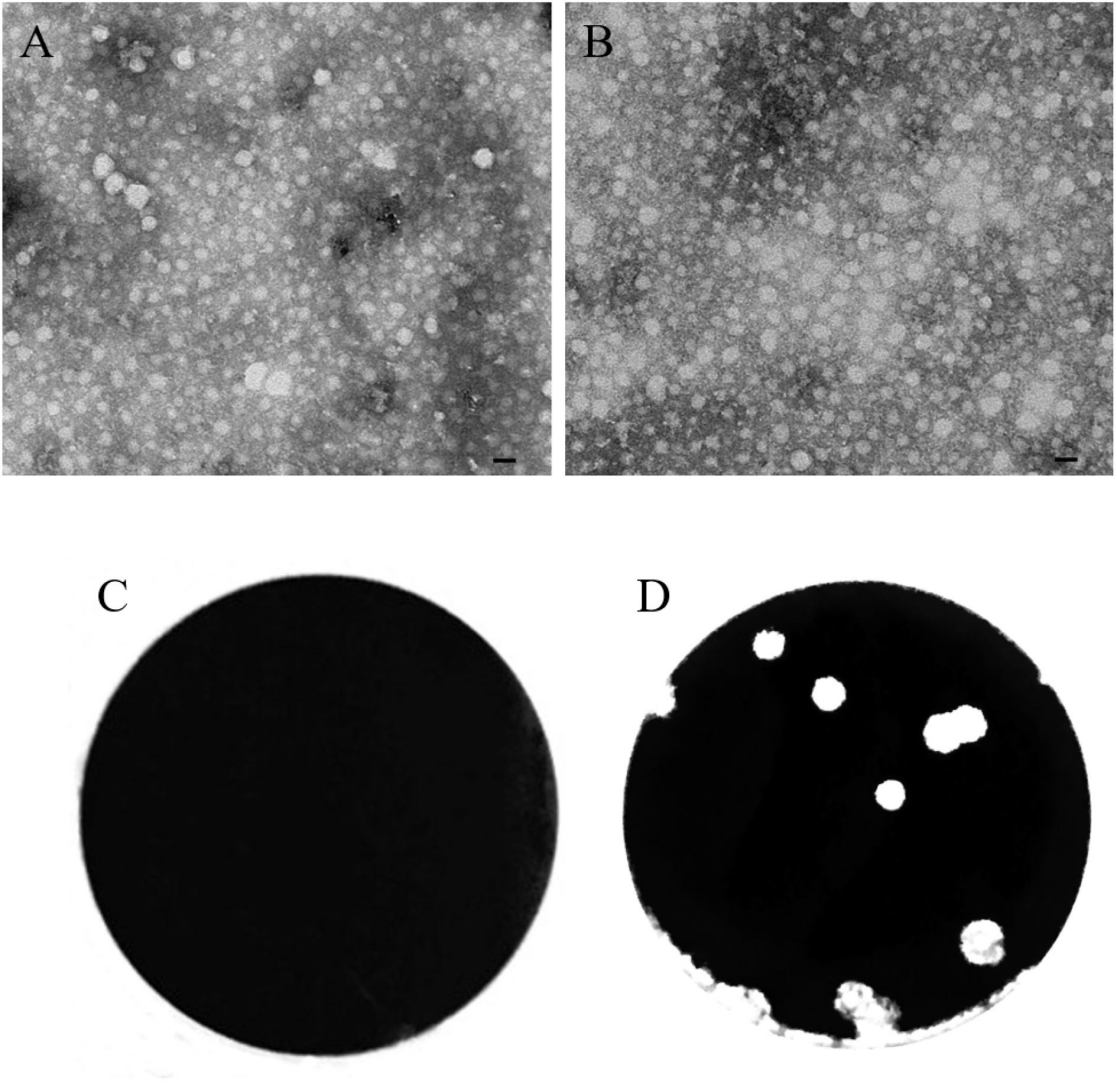
Characterization of the recombinant WM01ma-HA (H5) influenza virus. Comparison of the morphologies of WM01ma **(A)** and the recombinant WM01ma-HA (H5) influenza virus **(B)** by scanning electron microscopy (scale bar 100 nm), plaque formed by the recombinant WM01ma-HA (H5) influenza virus on wild-type MDCK cells **(C)** and MDCK cells stably expressing the NA protein **(D)**.

In the current study, the morphology of the WM01ma H9N2 influenza virus and the rescued WM01ma-HA(H5) was tested by using transmission electron microscopy (TEM). The results indicate that virion particles exhibit homogeneous morphologies of spheres with an average particle size of ~100 nm (Figures 3A,B). The plaque assay was also performed on MDCK cells and MDCK cells stably expressed the H9N2 NA protein. From the results, it is clear that WM01ma-HA (H5) replicated well on MDCK cells stably expressing the H9N2 NA protein but not in normal MDCK cells (Figures 3C,D).

**Figure 4:**
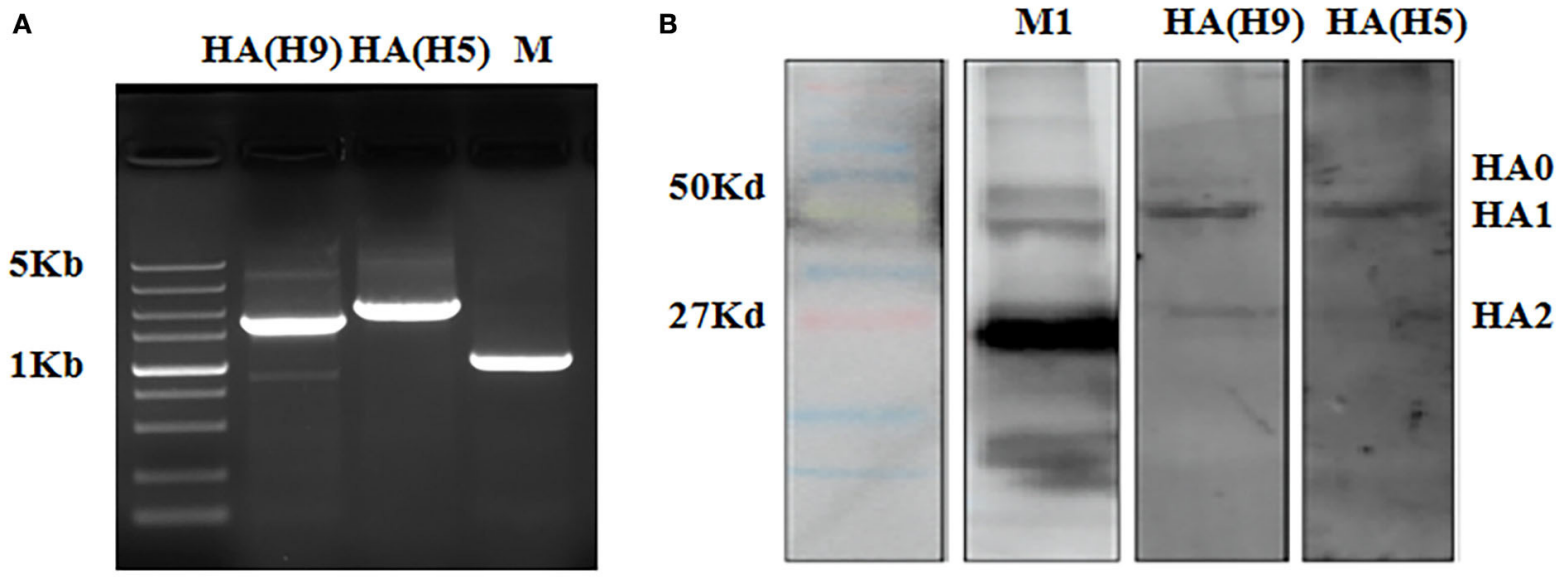
HAs of H9 and H5 were verified in the recombinant WM01ma-HA (H5) influenza virus by RT-PCR **(A)** and western blotting **(B)**.

**Figure 5:**
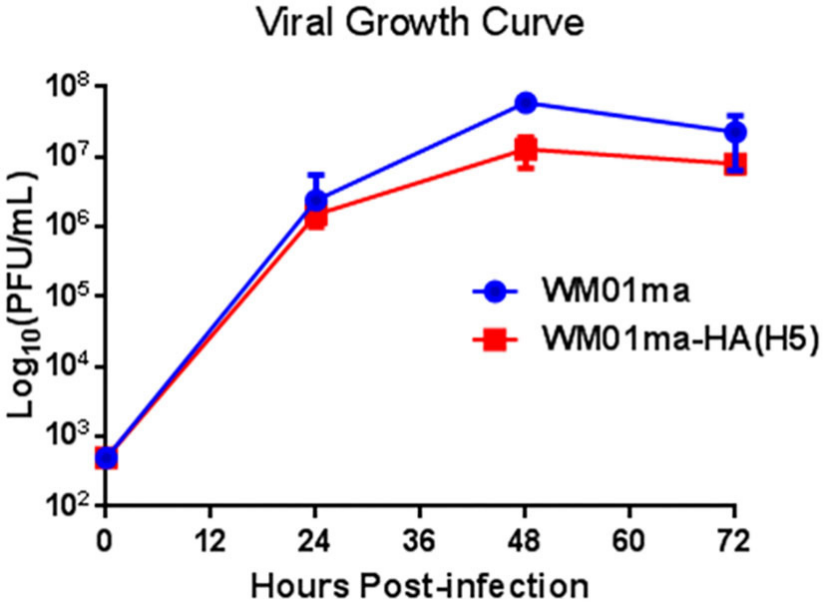
Growth curve of the recombinant WM01ma-HA (H5) influenza virus on MDCK cells stably expressing NA protein. The viral titers in each time point were calculated in triplicate. The values represent the means ± standard deviations (SD).

In order to verify that both H9 and H5 HAs are expressed on WM01ma-HA(H5) virus particles, gene segments of H9 and H5 HA were amplified by RT-PCR, and HA proteins were tested by Western blotting. The result evidenced the presence of H9 and H5 HA segments as they were detected in WM01ma-HA(H5) influenza virus (Figure 4A), and H9 and H5 HAs were concurrently presented in WM01ma-HA(H5) (Figure 4B). It was desired to know the discrepancy in the replication abilities of the WM01ma and WM01ma-HA(H5) viruses. A viral replication test was conducted in MDCK cells stably expressing the H9N2 NA protein. The results indicate that the WM01ma-HA(H5) virus exhibited reduced replication kinetics compared with that of the parental virus WM01ma with no significant difference (Figure 5). At 48-h postinfection, WM01ma and recombinant WM01ma-HA(H5) influenza virus attained the highest replication rate.

### Safety of the recombinant WM01ma-HA(H5) virus in mice

**Figure 6:**
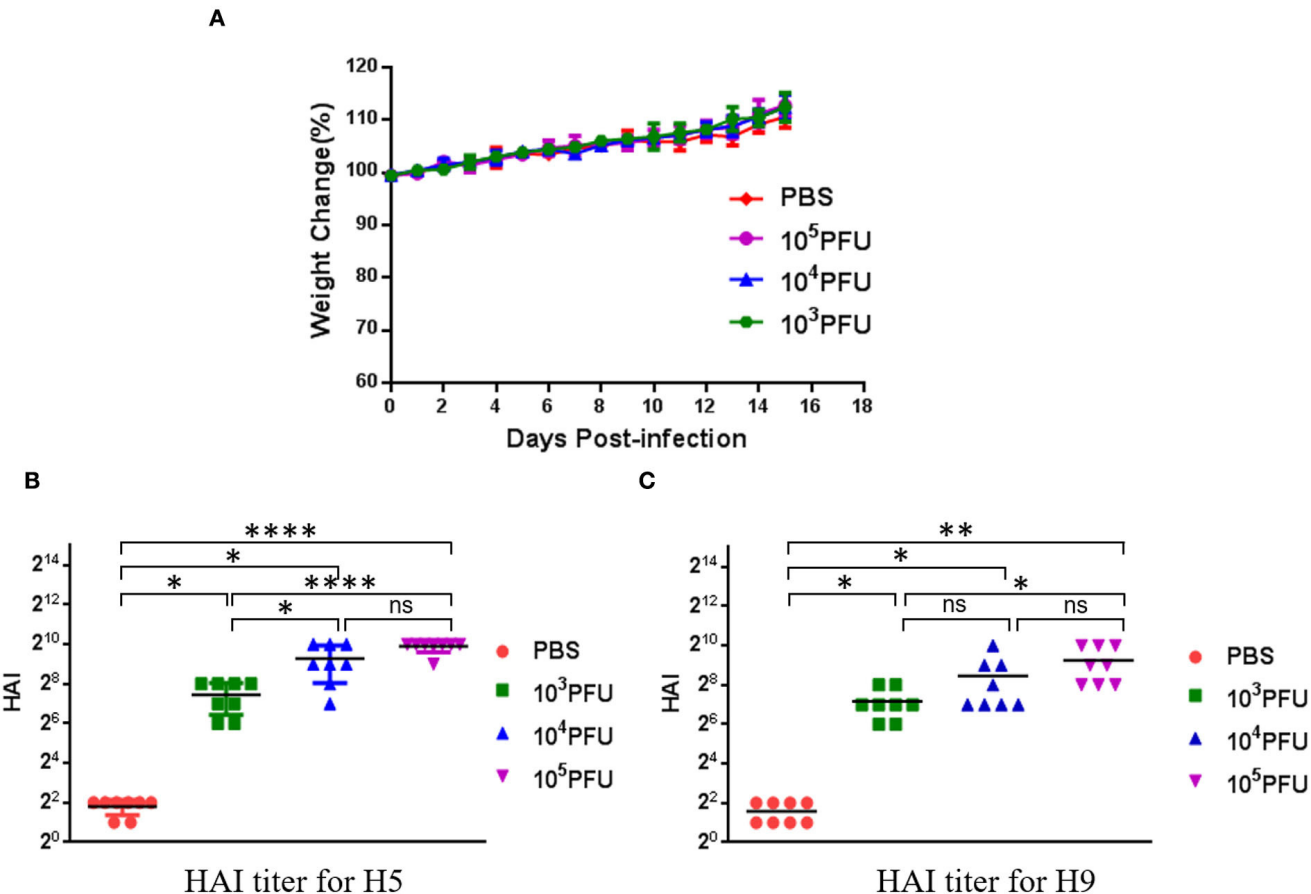
Evaluation of safety of the recombinant WM01ma-HA (H5) influenza virus as indicated by weight changes of experimental mice **(A)** and HAI titers against the HN021ma influenza virus **(B)** and the WM01ma influenza virus **(C)**. Each symbol represents an individual mouse, and the horizontal lines indicate mean values. The differences between the two groups were analyzed by a one-way ANOVA with Tukey's multiple-comparison test. ns indicates not significant. (*p* > 0.05), *(*p* ≤ 0.05), **(*p* ≤ 0.01), ****(*p* ≤ 0.0001).

To confirm the safety of the WM01ma-HA(H5) influenza virus in mice, three groups comprising of 8-week-old female BABL/c mice were inoculated intranasally with 10^3^, 10^4^, 10^5^ × PFU WM01ma-HA(H5) influenza virus, respectively, and one group was set as control. Mice of each group were monitored daily for 15 consecutive days for weight and illness (Figure 6A). As can be observed from the data, the weight of mice evenly increased over time and it indicated there was no adverse effect caused by inoculation with the WM01ma-HA(H5) influenza virus in all groups. The result suggested that the WM01ma-HA(H5) virus produced no harmful effect and stood safe *via* intranasal administration. Histopathological examination suggested that WM01ma-HA(H5) did not cause any pathological changes in lungs of mice (Supplementary Figure S2).

### WM01ma-HA(H5) influenza virus induced potent immune response and protection against H9N2 and H5N1 viruses in mice

To test the humoral immune response indicated by HAI titer by the replication-deficient WM01ma-HA(H5) influenza viruses, previously immunized mice were given a booster dose on day 15 with the identical dose of WM01ma-HA(H5) influenza viruses. Mice serum samples were collected on day 29 to determine the titers of HA antibodies against homologous H5N1 (HN021ma) (Figure 6B) and H9N2 (WM01ma) (Figure 6C) viruses. To further validate the humoral immune response contributes to the protection against homologous H5N1 and H9N2 influenza virus infection, antibody microneutralization assays were performed using MDCK cells. The results demonstrated that inoculation with WM01ma-HA(H5) elicits neutralizing serum antibody titers against H5N1(HN021ma) and H9N2 (WM01ma) influenza virus (Supplementary Figure S3). It showed immunization with WM01ma-HA(H5) influenza viruses induced high levels of HA antibodies against H9N2 and H5N1 avian influenza viruses.

**Figure 7:**
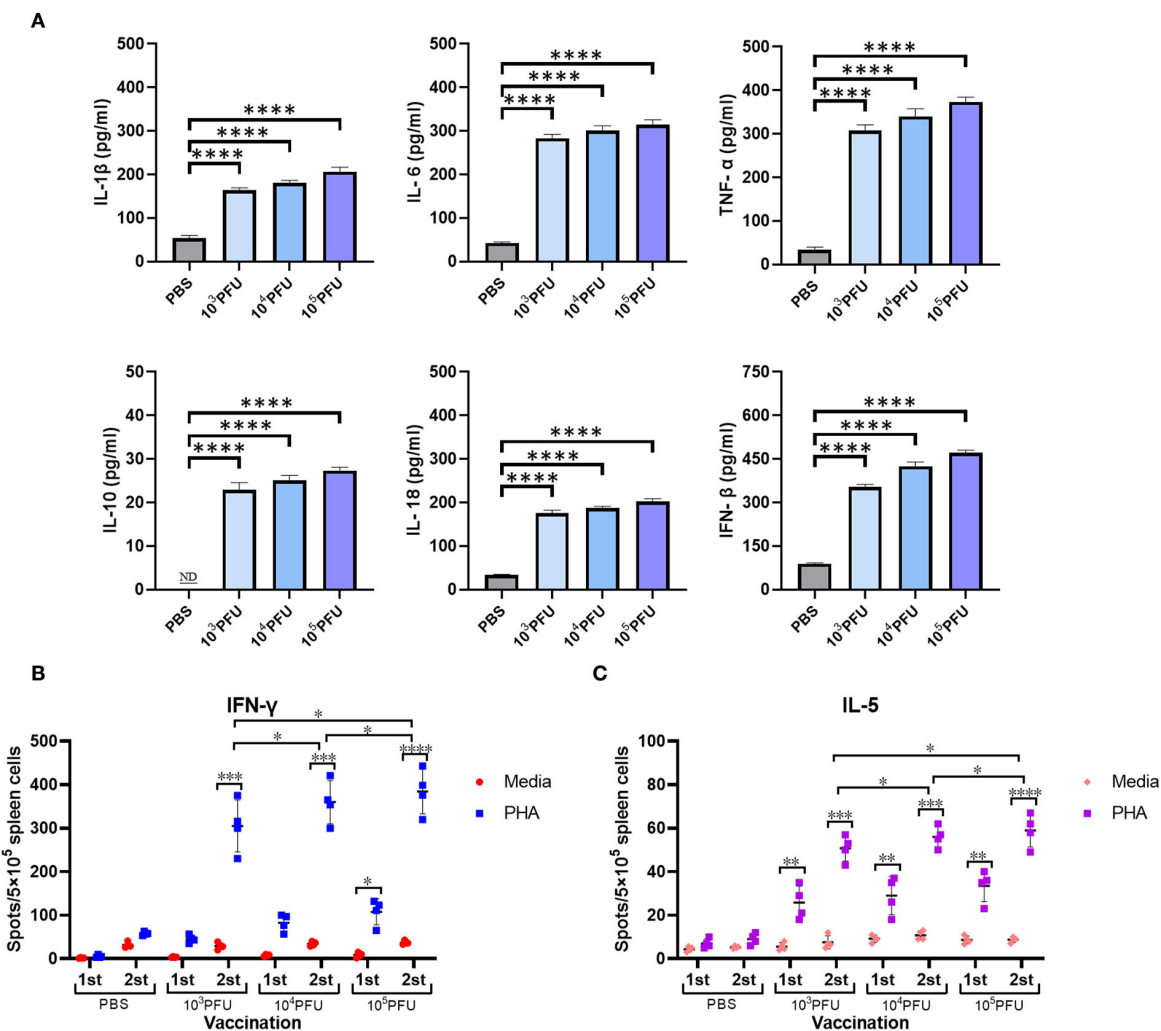
Cytokine secretion in intranasally vaccinated mice. Mice serum was collected at 3 days postinfection for analysis by ELISA to determine cytokine levels **(A)**. The number of antigen-specific splenocytes secreting IFN-γ **(B)** or IL-5 **(C)** was determined by ELISpot. ND, nondetected. Each sample was tested in triplicate. The values represent the means ± standard deviations (SD). The dots represent the individual mice. ns indicates not significant. (*p* > 0.05), *(*p* ≤ 0.05), **(*p* ≤ 0.01), ***(*p* ≤ 0.001),****(*p* ≤ 0.0001).

To determine the associated host immune activation and inflammation during the mouse vaccination with replication-deficient WM01ma-HA(H5) influenza viruses, four groups of 8-week-old female BABL/c mice were inoculated intranasally with PBS or 10^3^, 10^4^, 10^5^ × PFU WM01ma-HA(H5) influenza virus, respectively. Mice serum samples were collected on day 3 for analysis by ELISA assay to determine the cytokine levels. High levels of proinflammatory cytokine were detected in mice inoculated with the WM01ma-HA(H5) influenza virus compared with the PBS control group (*p* ≤ 0.0001) (Figure 7A).

Furthermore, we evaluated the antigen-specific IFN-γ and IL-5 secreting cells induced by WM01ma-HA(H5) vaccination. The mouse splenocytes were harvested after the prime (day 15) and the booster (day 29) vaccination from the PBS control group, 10^3^, 10^4^, 10^5^ × PFU WM01ma-HA(H5) vaccinated mice. The amount of secreting cells was measured by both the IFN-γ and IL-5 ELISpot assay. The IFN-γ levels remained low compared with the PBS control group after the first inoculation with WM01ma-HA(H5). However, the booster vaccination with WM01ma-HA(H5) induced a significantly higher number of the IFN-γ secreting cells compared with the mock-vaccinated PBS control group (Figure 7B). Concomitantly, the levels of IL-5 were still significantly increased compared with the PBS group, although not as high as IFN-γ (Figure 7C).

**Figure 8:**
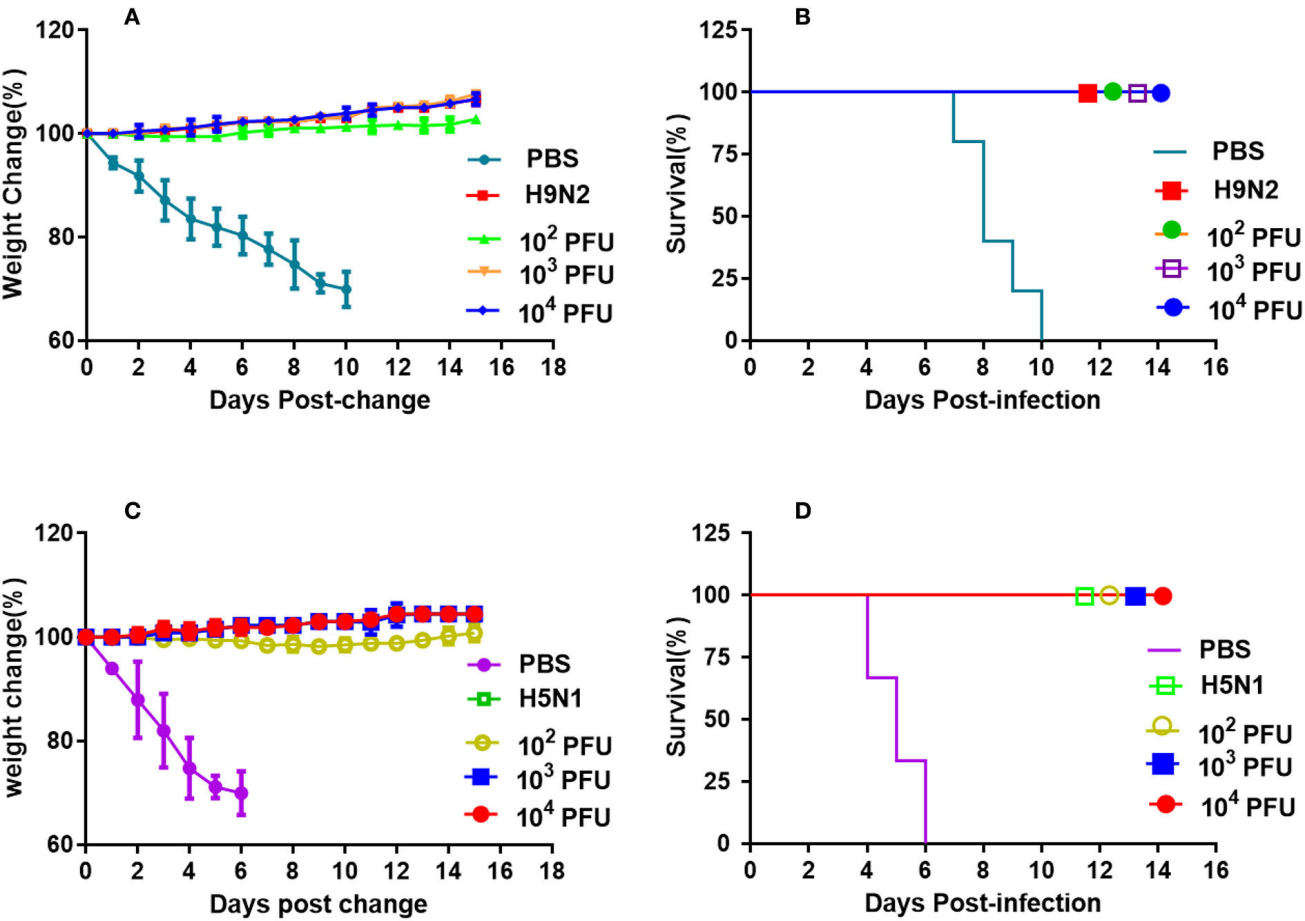
Vaccination with the recombinant WM01ma-HA (H5) virus conferred protection against HN021ma and WM01ma. **(A)** weight changes and **(B)** survival rates of mice vaccinated with the recombinant WM01ma-HA (H5) influenza virus and challenged with H9N2 (WM01ma) virus, **(C)** weight changes and **(D)** survival rates of mice vaccinated with the recombinant WM01ma-HA (H5) influenza virus and challenged with H5N1 (HN01ma) virus.

BABL/c mice of each group were inoculated intranasally with different doses of WM01ma-HA(H5) virus and challenged with 10 × MLD_50_ wild-type H9 or H5 subtype virus, respectively. After the mice were challenged, the vaccinated mice groups survived 100% and no weight losses were observed (Figure 8). However, animals of the positive control group challenged with the H9N2 influenza virus were all sacrificed in humanity at day 10 postinfection, and mice in the positive control group challenged with the H5N1 influenza virus died by day 6 because the weight loss is >25%.

**Figure 9:**
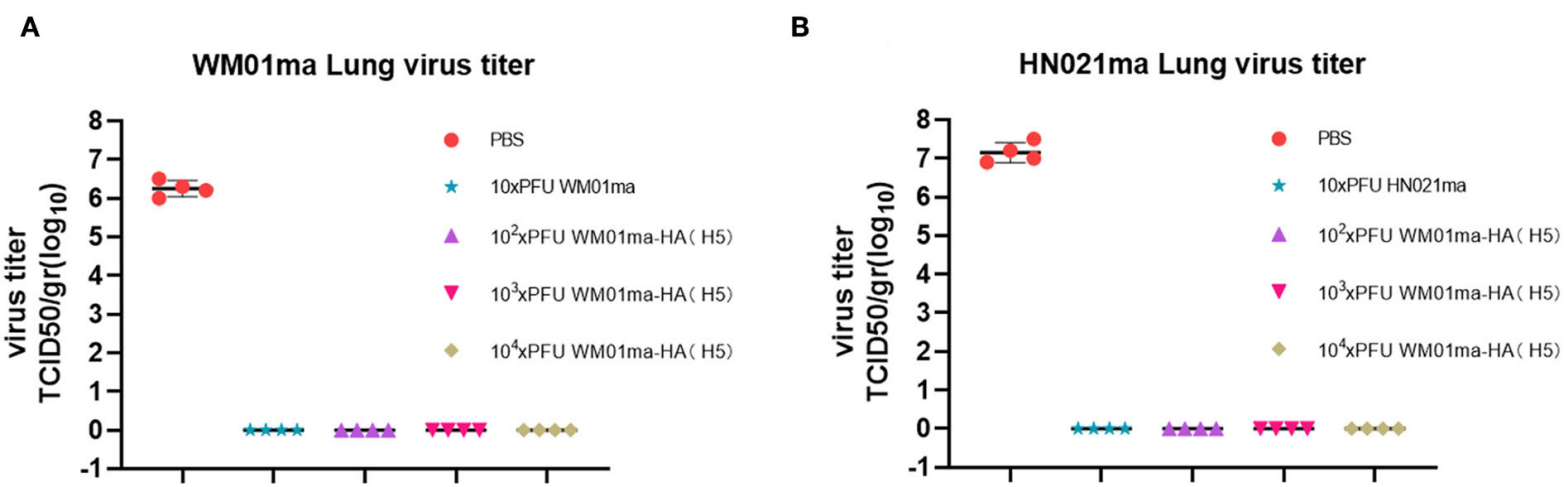
The viral titration in the lung tissues of WM01ma **(A)** or HN021ma **(B)** challenged mice at 4 d.p.i. Each experiment was performed in quadruplicate. The dots correspond to the values obtained from the individual mouse values and are represented as the means ± SD.

Four mice from each group were humanely sacrificed on 4 days postinfection (d.p.i) and the lungs were collected for virus isolation and titration. High virus titers could be detected in all unvaccinated mice (PBS mock-vaccinated) challenged with either WM01ma or HN021ma. No infectious virus could be detected in the lungs of WM01ma, HN021ma, or WM01ma-HA(H5) virus vaccinated mice (Figures 9A,B). It is consistent with survival and weight data. These results suggest that replication-deficient WM01ma-HA(H5) vaccination provides robust protection against the H9 or H5 subtype influenza virus with as low as 100 × PFU immunization.

## Discussion

The development of an effective vaccine against the avian influenza virus has been a core issue for many researchers in recent years. The scientific community anticipates another influenza pandemic will be devastating. Progress has been made in designing and developing a safe vaccine. However, the chances of viral strain mutation and emergence of the zoonotic, more virulent subtype cannot be excluded. It is very enlightening that no report till now surfaced highlighting human-to-human infection by H9N2 and H5Nx influenza viruses. Nevertheless, the influenza virus subtypes such as H9N2 and H5N1 flush from avian to human worsen the situation. In order to prevent and control the influenza virus led pandemic particularly when it acquires the capability for human-to-human transmission, an urgency appears to design and develop an effective vaccine that may confer solid immunity. In this study, a chimeric H9N2 replication-defective influenza virus was generated by reverse-genetic technology expressing both H9 and H5 HA in the genetic background of the mouse-adapted H9N2 IAV. This chimeric influenza virus is unproductive *in vitro* and *in vivo* unless functional NA is trans-complemented in a permissive cell line. The safety, immunogenicity, and protective efficacy of this replication-deficient recombinant WM01ma-HA(H5) influenza virus were studied and evaluated in mice. The results showed that this engineered virus was safe *in vivo* and capable of inducing a potent immune response against H9 and H5 IAVs.

Several studies reported the development of a trivalent or bivalent vaccine to prevent the infection caused by H9N2 and H5N1 influenza viruses, but these vaccines had certain limitations. Inactivated influenza viral vaccines developed as the result of these studies were applied in several clinical trials and data showed poor performance with salient feature low immunogenicity (Kandeil et al., 2016; Kim et al., 2017; Gomaa et al., 2019). Therefore, the above study was designed to find a way forward.

The research found that the 5' and 3' packaging signals of each vRNA segment of the influenza virus are crucial to assemble a progeny virion with newly synthesized vRNA segments. Previously, one group reported an approach for the generation of a reassortant chimeric replication-defective influenza virus which grew well *in vitro* through the addition of bacterial sialidase and provided protection against H5N1 and H7N9 infection in mice (Tian et al., 2021). Based on this information, it is workable to generate a genome-modified LAIV. Current inactivated influenza vaccines are suboptimal without inducing broad cross-protective activity (Houser and Subbarao, 2015). One of the key advantages of LAIVs over inactivated split or subunit influenza vaccines is their ability to induce both humoral and cellular immunity. Commercial LAIV (FluMist) is based on the cold-adaptation character which allows it to replicate at lower temperatures in the upper respiratory tract. However, replication is attenuated at the higher temperature in the lungs. Live attenuated influenza vaccines (LAIVs) have been shown to induce a significant amount of mucosal IgA but modest levels of serum IgG (Coelingh et al., 2014). The extraordinary potentiality of LAIVs to induce potent mucosal immunity is crucial for limiting virus transmission, especially when applied in response to a pandemic or a severe epidemic. However, this cold-adapted LAIV virus replicates efficiently in the upper respiratory tract of seronegative young children or those who have not been exposed to the previous infection with influenza viruses and shed at a comparatively high titer for a long period of time. Therefore, an improved more effective version of LAIV without suspected viral shedding and with extra potent properties inducing a full spectrum of protective immunity in the elderly population is necessary.

We successfully rescued a recombinant virus bearing a chimeric NA-HA(H5) segment in MDCK cells stably expressing the NA protein. The incorporation of the chimeric gene segment into the virion was confirmed by RT-PCR (Figure 4A) and the expression of the H9 and H5 HA was further confirmed by Western blotting (Figure 4B). *In vitro* study revealed that the safety of the vaccine is based on exogenous NA, as evidenced by the fact that the engineered virus was able to replicate and form visible plaques only in MDCK cells stably expressing the NA protein (Figures 3C,D). The replacement of the NA ORF with the H5 HA ORF resulted in the loss of the natural influenza virus neuraminidase enzymatic activity and reduced the replication potential of the chimeric recombinant virus WM01ma-(H5). One of the main functions of the NA is to help the viral particle to infect the host cell, without the NA activity, progeny virions will be stranded on the cellular membrane, unable to cleave sialic acids, and initiate a new replication cycle. The viral kinetics of the WM01ma-HA(H5) was slightly lower than that of the parental virus WM01ma but without significant differences (Figure 5). These results suggested that the virus could be a promising vaccine candidate with high-yield properties and might be applicable for future influenza vaccine development.

The pathogenicity of the replication-deficient virus WM01ma-(H5) was evaluated in mice. Clinical signs and histopathology results indicated that the replication-deficient virus WM01ma-(H5) was safe *via* intranasal administration (Figure 6A; Supplementary Figure S2). Furthermore, intranasal inoculation with the chimeric virions was able to induce neutralizing antibodies in serum against both the homologous WM01ma (H9N2) virus and HN021ma (H5N1) virus (Figures 6B,C; Supplementary Figure S3), suggesting that the presence of neutralizing antibodies is required to provide immunity against virus infection.

The continuous emergence of human infection with avian influenza A virus poses a persistent threat to human public health, therefore, we generated this replication-defective virus encoding two different subtypes of HA and desired it would alleviate public health stress by potential pandemic caused by H9N2 influenza virus as a vaccine candidate. In this study, a replication-defective influenza virus was generated, which is with certain unproductiveness unless functional NA is trans-complemented in the permissive cell line. This initial study evidences the biological safety and improved immune functionality of LAIVs both *in vitro* and in mice. In the following study, viral shedding and immune response after immunizations will be examined in mice and ferrets. Hopefully, it will serve as an effective vaccine candidate for the possible pandemic caused by the H9N2 or H5N1 influenza virus in humans.

## Materials and methods

### Cells and viruses

Madin–Darby canine kidney (MDCK) cells were maintained in Eagle's minimal essential medium (MEM) containing 10% fetal bovine serum (FBS; Biological Industries) and 1% Penicillin–Streptomycin Solution (PS; Biological Industries). Mouse-adapted A/Mink/Shandong/WM01/2014(H9N2)(WM01ma) and mouse-adapted A/wild duck/Hunan/021/2005 (H5N1)(HN021ma) were propagated at 37°C in the allantoic cavities of 9-day-old specific-pathogen-free (SPF) embryonated chicken eggs.

### Construction of the expression plasmid pd2-NA

The plasmid pd2EGFP-N1 and NA (WM01ma) coding DNA segments were enzymatically dissolved with restriction enzyme NheI and NotI, respectively, then the purified NA segment was inserted into the pd2EGFP-N1 vector. The linked product was transformed into *E.coli* DH5α competent cells and the target clones carrying designated plasmid pd2-NA were screened on the solid LB plates containing 50 μg/ml Kanamycin for 14 h at 37°C. The target plasmid pd2-NA was extracted and stored for further use.

### Establishment and verification of the MDCK stably expressing NA protein

3 × 105 MDCK cells were seeded in 35-mm dishes with MEM medium containing 10% fetal bovine serum (FBS) and 1% Penicillin–Streptomycin Solution (PS). On the following day, MDCK cells were electroporated with the pd2-NA following the protocol of the Nucleofector Kit for MDCK cells (Lonza, Cologne, Germany) (Wen et al., 2015). Briefly, MDCK cells were resuspended in a 0.1 ml solution I, and 5 μg plasmid pd2-NA was added into Solution I and mixed with MDCK cells. Then, the cell-plasmid mixture was transferred to an electroporation cuvette and treated on the Amaxa Nucleofector II machine (Lonza, Cologne, Germany) by using the A24 program. After being electroporated, cells were transferred to 6-well plates containing DMEM with 10% FBS. At 48-h post transmission, the cell monolayer was trypsinized, diluted 1:100, and seeded into 24-well plates containing DMEM with 5% FBS and the final concentration was 600 μg/ml G418. The medium was replaced every 3 days and cultured for 2 weeks. Surviving cell colonies were transferred to a 25 mm^2^ flask and continued to be cultured in the presence of G418. Finally, cells were scaled up and cultured for at least 10 passages to generate MDCK cells stably expressing the NA protein.

To verify the stable expression of the NA gene in MDCK-NA stable cells, RT-PCR and immunofluorescence assay (IFA) were performed. RNA of 5 × 10^6^ MDCK-NA cells was extracted with RNAiso Plus Reagent (Takara, Dalian, China) and was reverse transcribed with a PrimeScript^TM^II 1st Strand DNA Synthesis Kit (Takara, Dalian, China). The PCR product was separated on 1% agarose gel and data in the form of snaps were taken on Gel Doc Efficient Zeitgeist (Bio-Rad, Hercules, CA). The MDCK cells expressing the H9N2 NA protein were seeded in a 6-well plate fixed in 4% paraformaldehyde for 1 h, then permeabilized in PBS with 0.1% Triton X-100 for 30 min, and the cell samples were blocked in PBS containing 1% BSA for 1 h at room temperature. In the next step, the MDCK cells were incubated with H9N2 NA antibody at 4°C overnight. After three washes with PBST, the transfected MDCK cells were incubated with a secondary antibody (Goat anti-mouse IgG FITC antibody, BI). Finally, MDCK cells were observed and images were taken under a fluorescence microscope (ZEISS, AxioVert.A1).

### Construction of pHW-NAps-HA-NAps

The designated sequence NAps-HA-NAps contains the ORF of H5N1 HA flanked by the NA (H9N2) segment-specific packing signals at the 3' and 5' ends (202 nucleotides and 195 nucleotides, respectively) and was synthesized by GenScript. The translation initial codon and stop codon in the packaging signals of the NA (H9N2) gene segment were altered, then, the NAps-HA-NAps gene was amplified by PCR and cloned to vector pHW2000 (Hoffmann et al., 2001).

### Rescue of recombinant WM01ma-HA(H5) influenza virus

The recombinant virus was rescued with a standard reverse genetics method using the eight bidirectional plasmids system (Hoffmann et al., 2000). 3.5 × 10^5^ 293T cells and 3.5 × 10^5^ MDCK cells stably expressing NA protein were co-seeded in each well of a 6-well plate cultured in MEM medium supplemented with 10% FBS and 1% PS. On the following day, the medium was replaced by Opti-MEM (Gibco). The cells were co-transfected with eight plasmids including pHW-PB2, pHW-PB1, pHW-PA, pHW-HA, pHW-NP, pHW-M, pHW-NS, and pHW-NAps-HA-NAps mixing with Lipofectamine 2000 (Invitrogen). At 6-h post-transfection, the medium was replaced with 2 ml of fresh Opti-MEM. At 12-h post-transfection, TPCK-trypsin was added at the final concentration of 1 μg/ml into the medium. Supernatants containing WM01ma-HA(H5) influenza virus were harvested for 72 h after transfection. Then, the virus was propagated on MDCK cells stably expressing the NA protein for further use.

### Transmission electron microscopy

Recombinant WM01ma-HA(H5) virus was ultracentrifuged at 36,000 rotations per minute (rpm) for 2 h in a 20% glucose gradient. The pellet was then re-suspended in 30 μl PBS. The samples were adsorbed onto freshly glow discharged carbon-stabilized Parlodion-coated 400-mesh copper grids. The grids were then rinsed with buffer containing 20 mM Tris (pH 7.4) and 120 mM KCl, negatively stained with 1% phosphotungstic acid (pH 7.2), and then dried by blotting onto filter paper. Virions were visualized on a Hitachi H7600 transmission electron microscope (Hitachi High Technologies, USA, Schaumburg, IL) operating at 80 kV and digitally captured with a charge-coupled device (CCD) camera at 5-megapixel resolution (Core facility of Qingdao Agricultural University).

### Western blot

Recombinant WM01ma-HA(H5) virus was ultracentrifuged at 36,000 rpm for 2 h in a 20% glucose gradient. The pellet was re-suspended in 30 μl RIPA buffer and given an overnight incubation on ice. Lysed virus samples were loaded and separated on 10% SDS-PAGE, then transferred onto nitrocellulose membrane using a semi-dry trans-blot apparatus (Bio-Rad, Hercules, CA). The membranes were blocked in PBS with 1% Tween (PBST) and 5% non-fat milk for 1 h with anti-M1 monoclonal antibody (Abcam, catalog# ab25919), anti-HA(H9), or anti-HA(H5) multiclonal antibody (prepared and reserved in our lab) at 4°C overnight. After washing with PBST, the membranes were incubated with horseradish peroxidase (HRP)-conjugated goat anti-mouse IgG antibody (Abcam) at room temperature for 1 h. After being washed, the membranes were developed with chemiluminescent HRP substrate before imaging. Finally, the membranes were scanned in FUSION FX7(VILBER, French).

### Growth kinetics

Madin–Darby canine kidney (MDCK) cells stably expressing the NA protein were seeded in wells of 6-well plate. The confluent cell monolayer was infected with viruses in the multiplicity of infection (MOI) of 0.001. After 1 h of adsorption, the viral supernatant was removed, and the cells were washed two times with phosphate-buffered saline (PBS) and incubated in MEM containing 1 μg/ml TPCK-treated trypsin at 37°C. Supernatants were collected at indicated time points, and titers were determined by standard plaque assay in triplicate.

### Safety test, hemagglutination inhibition, and microneutralization assay in mice

Six–eight-week-old BALB/c mice were divided randomly into four groups with eight mice in each group. The virus was serially diluted in DPBS, and 50 μl was intranasally inoculated into mice anesthetized by isoflurane. After being challenged, mice were monitored for 15 days for clinical symptoms, weight loss, and death. At 15-day postinfection, the same doses were boosted. At 29-day postinfection, blood samples were collected for the HAI test.

Mouse sera were treated with a receptor-destroying enzyme to inactivate non-specific inhibitors (Denka Seiken Co. LTD.). Briefly, serum samples were 2-fold serially diluted in PBS in a 25 μl volume in 96-well V-shape microtiter plate, then an equal volume of 4-uint virus in 25 μl was added into each well. The plate was incubated at 37°C for 30 min. Subsequently, 25 μl of 0.8% (v/v) chicken red blood cell suspension was added to each well. The HAI titer was determined by the reciprocal dilution of the last well that contained non-agglutinated chicken red blood cells.

Neutralization antibody titers in inoculated mouse sera, reactive with HN021ma (H5N1) and WM01ma (H9N2) influenza viruses were detected in triplicate using a microneutralization assay, as described previously (Grund et al., 2011).

For histopathologic examination, mice were anesthetized with isoflurane and inoculation intranasally with PBS or 10^5^ × PFU WM01ma-HA(H5). Four days after inoculation, treated animals were sacrificed and the lungs were collected and fixed in 10% buffered formalin, processed, and stained with hematoxylin and eosin (H&E).

### Cytokine levels assay

For this study, 6–8-week-old BALB/c mice were divided randomly into four groups with eight mice in each group. The virus was serially diluted in DPBS, and 50 μl was intranasally inoculated into mice anesthetized by isoflurane. At 15-day postinfection, the same doses were boosted. At 3-day postinfection, serum samples were collected for cytokine levels test. Meanwhile, the mouse spleens were harvested after the prime (day 15) and the booster (day 29) vaccination, and IFN-γ and IL-5 secreting cells were measured by the ELISpot assay.

Cytokine levels of IL-1β, IL-6, TNF-α, IL-10, IL-18, and IFN-β in the peripheral blood for each mouse were determined by enzyme-linked immunosorbent assay (ELISA) kits according to the manufacturer's instructions (Invitrogen, USA; R&D Systems, Minneapolis, USA). The plates were read at 450 nm in the PowerWaveXS2 (BioTek, Instruments, India).

MultiScreen Filter Plates (Millipore, cat# MAIPS4510) were coated with either purified rat anti-mouse IFN-γ monoclonal antibody (BD Bioscience, cat# 551216) or purified rat anti-mouse IL-5 monoclonal antibody (BD Bioscience, cat# 554393) in coating buffer at a concentration of 5 μg/ml at 4°C overnight. Splenocyte cells were isolated from mice. After being washed, 5 × 10^5^ cells were seeded in each well, followed by stimulation with 10 μg/ml of either PHA or media at 37°C in 5% CO2 overnight. After stimulation of cells were washed five times with PBS containing 0.05% Tween 20 (PBST) and incubated with biotin-conjugated rat anti-mouse IFN-γ antibody or IL-5 antibody (2.0 μg/ml) at room temperature for 2 h. The plates were washed and incubated with horseradish peroxidase (HRP) conjugated streptavidin (BD Bioscience, cat# 555214) at room temperature for 1.5 h, washed with PBS, and then HRP substrate was added. Subsequently, the plates were kept at room temperature for 10 min in the dark, and the substrate was removed. After being washed with double-distilled water, the plates were dried overnight. Finally, spots were counted under the ELISpot reader (AID, Germany).

### Animal challenge experiments

Six–eight-week-old female BABL/c mice were assigned to five groups and 12 mice were assigned to each group. Mice in group 1 were set as positive control inoculated with PBS. Mice in group 2 as negative control were intranasally inoculated with 10 × PFU WM01ma, and groups 3–5 were intranasally inoculated with 10^2^, 10^3^, 10^4^ × PFU recombinant WM01ma-HA(H5) virus, respectively. On day 15 post-immunization, mice were infected with 10 × MLD_50_ WM01ma and monitored for 15 consecutive days for weight and clinical symptoms. Another set experiment was performed, the negative control was intranasally inoculated with 10 × PFU HN021ma, and mice were infected with 10 × MLD_50_ HN021ma on post-immunization day 15.

Four mice from each group were humanely sacrificed on 4 days postinfection (d.p.i) and their lung tissues were collected, weighed, and homogenized in 1 ml of cold PBS under sterile conditions, centrifuged at 12,000 rpm for 10 min at 4°C. Virus titers in the homogenized supernatant were measured by the 50% tissue culture infectious dose (TCID_50_) assay on MDCK cells. Madin–Darby canine kidney (MDCK) cells were seeded in 96-well plates at a density of 1.0 × 10^4^ cells/well and incubated at 37°C overnight. The homogenized supernatants were diluted 10 × fold serially. The MDCK cells were inoculated with a 100 μl diluted supernatant sample. After 3 days of incubation at 37°C, the TCID_50_ was determined using the Reed and Muench method.

### Statistical analysis

Comparisons between groups were performed by using a nonparametric one-way ANOVA with Tukey's multiple comparison test and Fisher's exact test, and survival rates were analyzed using the log-rank test. The analyses were performed using GraphPad Prism version 6.0 for Windows (GraphPad Software). *P*-values < 0.05 were considered to be significant.

## Data availability statement

The original contributions presented in the study are included in the article/Supplementary material, further inquiries can be directed to the corresponding author/s.

## Ethics statement

The animal study was reviewed and approved by the Institutional Animal Care and Use Committee at Qingdao Agricultural University (approval reference number # 20190020).

## Author contributions

WR, SP, and MZ conducted all experiments. LJ and HL collected samples. WJ and JL wrote the manuscript. YY and MH revised the manuscript. All authors have read and agreed to the published version of the manuscript.

## Funding

This study was supported by the National Natural Science Foundation of China (General Program, 32072837), Nanjing Important Science and Technology Specific Projects (2021-11005), Key Research and Development Program of the Department of Health of Jiangsu (ZDB2020036), and Key R&D Program of Jiangsu Province (Social Development) (BE2021603).

## Conflict of interest

The authors declare that the research was conducted in the absence of any commercial or financial relationships that could be construed as a potential conflict of interest.

## Publisher's note

All claims expressed in this article are solely those of the authors and do not necessarily represent those of their affiliated organizations, or those of the publisher, the editors and the reviewers. Any product that may be evaluated in this article, or claim that may be made by its manufacturer, is not guaranteed or endorsed by the publisher.
