# Supplementary material for: A replication-deficient H9N2 influenza virus carrying H5 hemagglutinin conferred protection against H9N2 and H5N1 influenza viruses in mice
Source: Front Microbiol. 2022 Nov 15;13:1042916. doi: 10.3389/fmicb.2022.1042916 (PMC9705590; doi:10.3389/fmicb.2022.1042916)
Supplement: Supplementary file 1 [file Data_Sheet_1.docx]

Supplementary Information


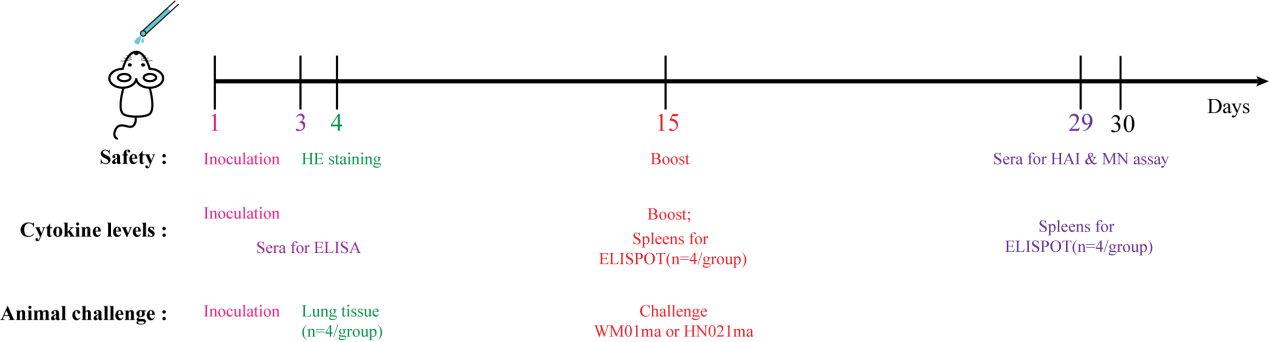


**Figure S1:** **Schedule of mouse immunization, sample collection and challenge**. BALB/c mice (n=8, each group) were intranasally inoculated on day 1 and boosted on day 15 with 10^3^,10^4^,10^5^xPFU of WM01ma-HA(H5) or PBS. At 3 days post-infection, serum samples were collected for cytokine levels test by ELISA. At 29 day post-infection, blood samples were collected for HAI and MN tests. After the prime (day 15) and the booster (day 29) inoculation from the WM01ma-HA(H5) or PBS inoculated mice. The splenocytes were isolated and the antigen-specific T cell responses were measured by the IFN-γ or IL-5 ELISpot assay. In the animal challenge experiments, BALB/c mice (n=8, each group) were intranasally inoculated on day 1 with 10^2^,10^3^,10^4^xPFU of WM01ma-HA(H5) virus, PBS or negative control (10xPFU WM01ma or HN021ma) and challenged with 10xMLD50 WM01ma or HN021ma virus on post-immunization day 15, respectively. Four days post challenge, four mice per group were euthanized for collecting lung tissue and the Virus titers were measured by TCID50.


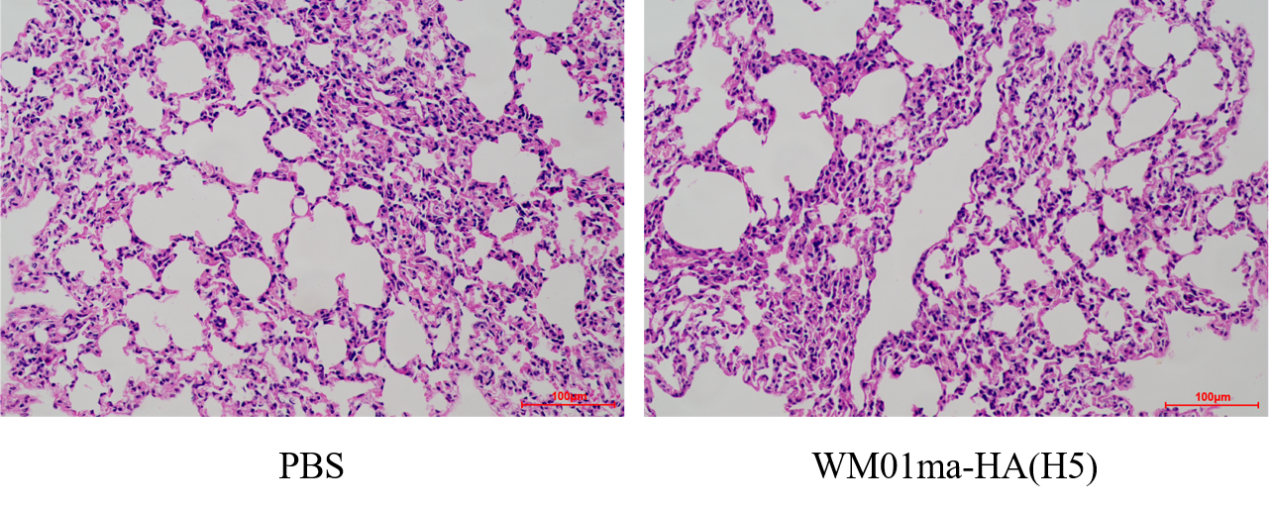


**Figure S2: Histopathological changes in lungs of mice inoculated with replication-deficient WM01ma-HA(H5) viruses.** BALB/c mice were inoculated intranasally with PBS or 10^5^xPFU WM01ma-HA(H5). The animals were sacrificed on 4th day after inoculation, and the lungs were collected for H&E staining and histopathological analysis. WM01ma-HA(H5) caused no pathological changes were observed compared to PBS group.

**
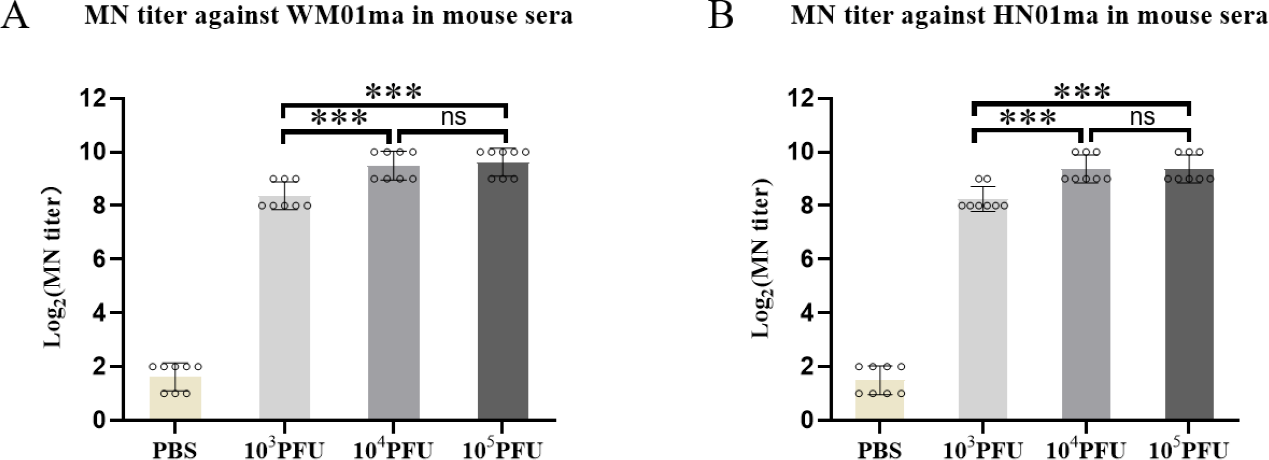
**

**Figure S3: Microneutralization (MN) titers against WM01ma (H9N2) and HN021ma (H5N1) influenza virus in sera from vaccinated mice.**

Each circle represents an individual mouse (n=8, each group), and the values represent the means±standard deviations (SD). Each sample was tested in triplicate. The differences between two groups were analyzed by a one-way ANOVA with Tukey’s multiple-comparison test. ns (p>0.05), *** (p≤0.001)).
